# Risk factors for small bowel bleeding in an overt gastrointestinal bleeding presentation after negative upper and lower endoscopy

**DOI:** 10.1371/journal.pone.0212509

**Published:** 2019-02-20

**Authors:** Dejan Micic, John N. Gaetano, Neha Nigam, Matthew Peller, Vijaya L. Rao, Carol Semrad, Adam C. Stein, Sonia S. Kupfer

**Affiliations:** 1 University of Chicago Medicine, Department of Internal Medicine, Section of Gastroenterology, Hepatology and Nutrition, Chicago, IL,United States of America; 2 Northwestern Medicine, Department of Internal Medicine, Chicago, IL, United States of America; 3 Northwestern Medicine, Department of Internal Medicine, Division of Gastroenterology and Hepatology, Chicago, IL, United States of America; Cleveland Clinic, UNITED STATES

## Abstract

**Introduction:**

A small bowel source is suspected when evaluation of overt gastrointestinal (GI) bleeding with upper and lower endoscopy is negative. Video capsule endoscopy (VCE) is the recommended next diagnostic test for small bowel bleeding sources. However, clinical or endoscopic predictive factors for small bowel bleeding in the setting of an overt bleeding presentation are unknown. We aimed to define predictive factors for positive VCE among individuals presenting with overt bleeding and a suspected small bowel source.

**Methods:**

We included consecutive inpatient VCE performed between September 1, 2012 to September 1, 2015 for melena or hematochezia at two tertiary centers. All patients had EGD and colonoscopy performed prior to VCE. Patient demographics, medication use, and endoscopic findings were retrospectively recorded. VCE findings were graded based on the P0-P2 grading system. The primary outcome of interest was a positive (P2) VCE. The secondary outcome of interest was the performance of a therapeutic intervention. Data were analyzed with the Fisher exact test for dichotomous variables and logistic regression.

**Results:**

Two hundred forty-three VCE were reviewed, and 117 were included in the final analysis. A positive VCE (P2) was identified in 35 (29.9%) cases. In univariate analysis, a positive VCE was inversely associated with presence of diverticula on preceding colonoscopy (OR: 0.44, 95% CI: 0.2–0.99), while identification of blood on terminal ileal examination was associated with a positive VCE (OR: 5.18, 95% CI: 1.51–17.76). In multivariate analysis, only blood identified on terminal ileal examination remained a significant risk factor for positive VCE (OR: 6.13, 95% CI: 1.57–23.81). Blood on terminal ileal examination was also predictive of therapeutic intervention in both univariate (OR: 4.46, 95% CI: 1.3–15.2) and multivariate analysis (OR: 5.04, 95% CI: 1.25–20.32).

**Conclusion:**

Among patients presenting with overt bleeding but negative upper and lower endoscopy, the presence of blood on examination of the terminal ileum is strongly associated with a small bowel bleeding source as well as with small bowel therapeutic intervention. Presence of diverticula on colonoscopy is inversely associated with a positive VCE and therapeutic intervention in univariate analysis.

## Introduction

Acute lower gastrointestinal bleeding (LGIB) accounts for ~20% of gastrointestinal bleeding (GIB) presentations, whereas small bowel bleeding accounts for ~5–10% of GIB presentations [1, 2]. Clinical features can be used to help differentiate rapid upper GIB (UGIB) from LGIB to include the presence of hemodynamic instability, an elevated BUN-to-creatinine ratio and passage of blood clots [2]. However, differentiating small bowel bleeding from LGIB can be clinically challenging and requires identification of predictive factors related to small bowel bleeding in order to improve the clinical utilization of diagnostic testing [3]. Improved prediction of small bowel bleeding is important because small bowel bleeding has been associated with higher resource utilization compared to acute UGIB or LGIB [3].

Video capsule endoscopy (VCE) is now recommended as the first-line procedure for a small bowel evaluation after upper and lower sources of GIB have been excluded by endoscopy and colonoscopy [1]. Identified risk factors for positive VCE include presence of overt bleeding, inpatient status, male sex and age over 60 years [4]. However, additional risk factors associated with positive VCE among those with an overt bleeding presentation and inpatient evaluation have not been identified. Specifically, the presence of non-bleeding colonic diverticula, a common finding on colonoscopy performed for overt GI bleeding, has not been examined as a predictor of a small bowel bleeding source. Therefore, we aimed to define predictive factors for small bowel bleeding in hospitalized patients with overt GIB who do not have an obvious source of bleeding on upper endoscopy or colonoscopy.

## Methods

### Inclusion and exclusion criteria

We performed a retrospective analysis of inpatient VCE procedures performed at two tertiary centers identified through query of the electronic data warehouse (EDW) using the CPT code for VCE 91111 or data reporting system (ProVation®, ProVation Medical, Minneapolis, MN) between 9/1/2012 and 9/1/2015. Inclusion criteria included available upper and lower endoscopy reports, negative findings on upper and lower endoscopy and a presentation with overt bleeding as documented in the medical record (melena, hematochezia or maroon stool). An upper endoscopy was considered to be negative if a probable bleeding source was not found. Probable bleeding sources included: the presence of fresh or old blood, varices with stigmata of hemorrhage, Mallory-Weiss tears, severe reflux esophagitis (Los Angeles grade C or D), ulcers with stigmata of hemorrhage, angiodysplasias or Dieulafoy’s lesions requiring treatment, or tumors with stigmata of hemorrhage [5]. A colonoscopy was considered to be negative if it lacked inflammatory bowel disease, angiodysplasias requiring treatment, or ulcers or diverticula with active or stigmata of hemorrhage. Studies with an incomplete examination of the small bowel, those performed > 7 days after admission or repeated procedures were excluded from the analysis. This study was approved by the Institutional Review Boards at the University of Chicago (IRB17-1323) and Northwestern University (STU00202278).

### VCE

All VCEs were performed using the Given Imaging PillCam SB2/SB3 (Given Imaging, Yokne’am Illit, Israel) and graded using the P0-P2 categorical system as described previously [6]. Briefly, P2 lesions were those with a high potential for bleeding, and included typical angioectasias, ulcerations, masses or varices. P1 lesions were those with an uncertain hemorrhagic potential and included red spots or small isolated erosions. P0 lesions were those with no potential for bleeding, and included submucosal veins, diverticula without blood and nodules without a mucosal break. Fresh blood in the small bowel on VCE examination was considered a P2 study. Only VCE examinations with positive findings in the small bowel were considered positive. Purgative preparation or simethicone was not routinely used.

### Factors considered in risk analysis

Endoscopic factors obtained from chart review included the presence of non-bleeding diverticula on colonoscopy, the presence of non-bleeding diverticula proximal to the splenic flexure (proximal diverticula) and intubation of the terminal ileum with note of presence or absence of blood. Medications at the time of patient admission were recorded for the use of antiplatelet therapies, anticoagulant medication and non-steroidal anti-inflammatory drugs (NSAID). The presence of a left-ventricular assist device (LVAD) at the time of VCE was recorded in chart review. Additional variables collected from the EDW included hemoglobin, platelet count, blood urea nitrogen (BUN), creatinine and international normalized ratio (INR) at the time of VCE and Charlson-Deyo comorbidity score for the admission [7, 8]. Therapeutic interventions as a secondary outcome of interest included the performance of a push enteroscopy, balloon-assisted enteroscopy or surgical intervention following the VCE.

### Statistical analysis

The primary outcome of interest was identification of risk factors for a positive VCE (P2 lesion). For the purposes of analysis, three individuals presenting with maroon stool were categorized as hematochezia. The Fisher’s exact test was used to compare categorical variables. Logistic regression was used to determine an association between the dependent outcomes, positive VCE and therapeutic intervention, and independent variables. Associations were described with odds ratios with 95% confidence intervals (CI). Factors significant in univariate analysis with a p-value < 0.1 were included in multivariate analysis. A two-sided p-value ≤ 0.05 was considered statistically significant. Statistical analysis was conducted using JMP 13.1.0 (SAS Institute, Inc., Cary, NC).

## Results

**Fig 1:**
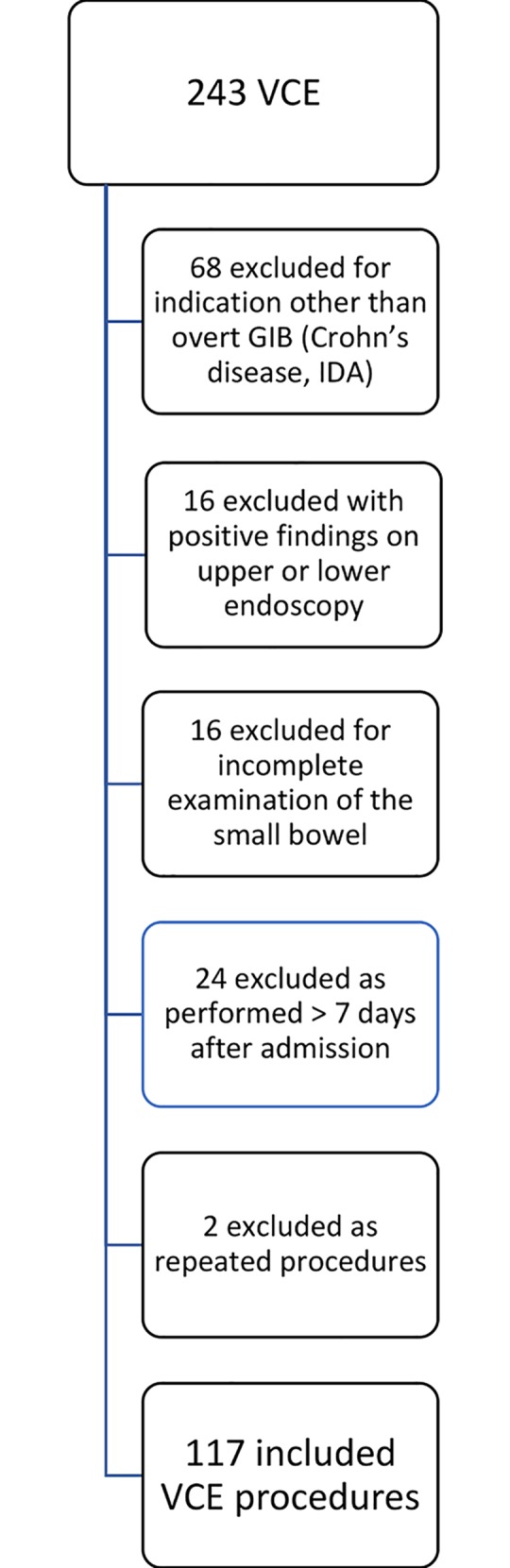
Flowchart of included and excluded examinations. GIB = gastrointestinal bleeding; IDA = iron deficiency anemia; VCE = video capsule endoscopy.

A total of 243 VCE procedures with available upper and lower endoscopy reports were identified. Studies performed for indications other than overt bleeding (n = 68), those with an incomplete examination of the small bowel (n = 16), those performed > 7 days after admission (n = 24) or repeated procedures (n = 2) were excluded (**Fig 1**). One hundred seventeen VCE procedures were included in the final analysis. The median time from admission to VCE performance was 2.2 days (IQR: 1–4) and presentation with melena accounted for the majority of overt bleeding events (63.2%). Left-ventricular assist device was present in 23 (19.7%) patients and a positive VCE (P2) was identified in 35 (29.9%) cases. All individuals had negative evaluations on upper and lower endoscopy, while only 64 (54.7%) had intubation of the terminal ileum as part of the diagnostic evaluation (**Table 1**). Thirty-two individuals underwent subsequent therapeutic procedures of the small bowel including surgery (n = 1) or double-balloon enteroscopy (n = 31).

**Table 1:** Demographic features of included patients and video capsule examinations.

	VCE (n = 117)
**Demographics (n, %)**	
Age (yr, median, IQR)	67 (59–77)
Male Sex	62 (52.9%)
Charlson score (mean, SD)	3.1 (2.4)
Time from admission to VCE (days, median, IQR)	2.2 (1–4)
Melena Indication	74 (63.2%)
Hematochezia Indication	43 (36.8%)
Left Ventricular Assist Device (LVAD) present	23 (19.7%)
**Video capsule (n, %)**	
Positive (P2) VCE	35 (29.9%)
Angioectasia	13 (11.1%)
Blood on VCE	39 (33.3%)
Ulceration	8 (6.8%)
Mass	5 (4.3%)
**Colonoscopy (n, %)**	
Diverticula on colonoscopy	73 (62.3%)
Proximal diverticula	49 (41.8%)
Terminal ileum intubated	64 (54.7%)
Blood in small bowel on colonoscopy	15 (12.8%)
**Medication use on admission (n, %)**	
Aspirin	58 (49.5%)
Coumadin	36 (30.7%)
Other antiplatelet	10 (8.5%)
Other anticoagulant	9 (7.6%)
Nonsteroidal anti-inflammatory drug	12 (10.3)
**Laboratory values**	
Hemoglobin, g/dL, mean (SD), n = 114	8.7 (1.3)
Platelets, (10^9^/L), mean (SD), n = 113	202 (82.9)
BUN, mg/dL, mean (SD), n = 109	22 (17.5)
Creatinine, mg/dL, mean (SD), n = 109	1.66 (1.7)
INR, mean (SD), n = 75	1.46 (0.57)

(BUN: blood urea nitrogen, INR: international normalized ratio, IQR: interquartile range)

### Risk factors for positive VCE

Risk factors associated with positive VCE are shown in **Table 2**. In univariate analysis, a positive VCE was less likely when diverticula were identified on preceding colonoscopy (OR: 0.44, 95% CI: 0.2–0.99, p = 0.046). Proximal diverticula were similarly inversely related to a positive VCE examination (OR: 0.44, 95% CI: 0.19–1.03, p = 0.06); though this result only trended toward statistical significance. In the setting of diverticula on preceding colonoscopy, 17/73 (23.3%) VCE examinations were positive as compared to 18/44 (40.9%) positive VCE examinations when diverticula were absent (p = 0.06).

**Table 2:** Predictive factors for positive video capsule endoscopy in overt gastrointestinal bleeding.

		Univariate			Multivariate
	Positive VCE (n = 35)	Negative VCE (n = 82)	OR (95% CI)	*p*-value	OR (95% CI)
**Demographics**					
Age (yr, mean, SD)	68.4 (11.3)	64.9 (15.4)	1.01 (0.99–1.05)	0.238	
Male Sex	20 (57.1)	42 (51.2)	1.26 (0.57–2.82)	0.557	
Charlson score (mean, SD)	3.5 (2.3)	2.9 (2.4)	1.1 (0.93–1.3)	0.255	
Hematochezia Indication	11 (31.4)	32 (39)	ref		
Melena Indication	24 (68.6)	50 (61)	1.4 (0.6–3.24)	0.436	
Left Ventricular Assist Device (LVAD) present	10 (28.6)	13 (15.9)	2.12 (0.83–5.45)	0.118	
**Colonoscopy**					
Diverticula on colonoscopy	**17 (48.6)**	**56 (68.3)**	**0.44 (0.2–0.99)**	**0.046**	1.08 (0.2–5.87)
Proximal diverticula	10 (28.6)	39 (47.6)	0.44 (0.19–1.03)	0.060	0.44 (0.07–2.72)
Blood in terminal ileum on colonoscopy	**9 (45)**	**6 (13.6)**	**5.18 (1.51–17.76)**	**0.009**	**6.13 (1.57–23.81)**
Blood in the colon lumen	14 (40)	21 (25.6)	1.94 (0.84–4.48)	0.122	
**Medication use on admission**					
Aspirin	19 (54.3)	39 (47.6)	1.31 (0.59–2.9)	0.506	
Coumadin	14 (40)	22 (26.8)	1.82 (0.79–4.19)	0.160	
Nonsteroidal anti-inflammatory drug	2 (5.7)	10 (12.2)	0.45 (0.09–2.1)	0.302	
**Laboratory values**					
Hemoglobin, g/dL, mean (SD)	8.4 (1.4)	8.9 (1.3)	0.75 (0.55–1.04)	0.084	0.86 (0.5–1.48)
Platelets, (10^9^/L), mean (SD)	198 (67)	204 (89)	1 (0.99–1)	0.758	
BUN, mg/dL, mean (SD)	26.5 (20)	19.9 (15.9)	1.02 (1–1.04)	0.080	1.01 (0.97–1.05)
Creatinine, mg/dL, mean (SD)	1.57 (1.3)	1.7 (2)	0.96 (0.75–1.22)	0.722	
INR, mean (SD)	1.57 (0.73)	1.41 (0.47)	1.61 (0.71–3.68)	0.255	

(BUN: blood urea nitrogen, INR: international normalized ratio)

Among individuals that underwent intubation of the terminal ileum (n = 64), 15 (23%) had blood visualized in the small bowel. Identification of blood on terminal ileal examination was associated with a positive VCE (OR: 5.18, 95% CI: 1.51–17.76, p = 0.009). Nine of 15 (60%) individuals with blood identified in the terminal ileum had a positive VCE as compared to 11 of 49 (22.45%) individuals without blood on terminal ileal examination (p = 0.01). Blood urea nitrogen trended to higher values in individuals with a positive VCE (OR: 1.02, 95% CI: 1–1.04, p = 0.08) while hemoglobin trended to lower values in individuals with a positive VCE (OR: 0.75, 95% CI: 0.55–1.04, p = 0.08).

In multivariate analysis, only blood identified on terminal ileal examination remained a significant risk factor for positive VCE (OR: 6.13, 95% CI: 1.57–23.81). When excluding individuals with an LVAD (n = 23), blood on terminal ileal intubation remained associated with a positive VCE (OR: 5.4, 95% CI: 1.31–22.18, p = 0.019) and the presence of proximal diverticula on colonoscopy was inversely associated a positive VCE (OR: 0.33, 95% CI: 0.12–0.91, p = 0.033) in univariate analysis.

### Risk factors for performance of therapeutic intervention

Thirty-two (27%) individuals underwent a therapeutic procedure to the small bowel of which 28 (87.5%) had a positive VCE. Median time from VCE to intervention was 3 (IQR: 2–13) days. Findings on therapeutic procedures are listed in **Table 3**. Similar predictors of a therapeutic procedure were identified (**Table 4**). In univariate analysis, diverticula on preceding colonoscopy, as well as proximal diverticula were inversely associated with the performance of a therapeutic procedure (diverticula: OR: 0.29, 95% CI 0.12–0.66; proximal diverticula: OR: 0.35, 95% CI: 0.14–0.89). Blood in the small bowel lumen on terminal ileal examination remained a significant predictor of a therapeutic procedure both in univariate (OR: 4.46, 95% CI: 1.3–15.2) and multivariate analysis (OR: 5.04, 95% CI: 1.25–20.32). When included in separate multivariate models with blood on terminal ileal examination, the presence of diverticula (OR: 0.22, 95% CI: 0.06–0.85) but not proximal diverticula (OR: 0.24, 95% CI: 0.06–1.01) remained significant independent negative predictors of a therapeutic procedure.

**Table 3:** Findings on attempted therapeutic small bowel examinations.

Finding	Number
Angioectasia	12
Streaming lesion	5
Negative examination	5
Ulcerated anastomosis/stenosis	2
Polyp/mass lesion	2
Ulcerations	1
Clot	1
Varices	1
Diverticula	1
Submucosal lesions	1
Incomplete examination	1

**Table 4:** Predictive factors for performance of therapeutic intervention for small bowel bleeding.

		Univariate			Multivariate
	Therapeutic intervention (n = 32)	No therapeutic intervention (n = 85)	OR (95% CI)	*p*-value	OR (95% CI)
**Demographics**					
Age (yr, mean, SD)	66.6 (11.5)	65.7 (15.3)	1 (0.98–1.03)	0.771	
Male Sex	20 (62.5)	42 (49.4)	1.71 (0.74–3.92)	0.208	
Charlson score (mean, SD)	3.53 (2.5)	2.9 (2.3)	1.12 (0.94–1.33)	0.202	
Hematochezia Indication	10 (31.3)	33 (38.8)	ref		
Melena Indication	22 (68.8)	52 (61.2)	1.4 (0.59–3.32)	0.449	
Left Ventricular Assist Device (LVAD) present	9 (28.1)	14 (16.5)	1.98 (0.76–5.18)	0.162	
**Colonoscopy**					
Diverticula on colonoscopy	**13 (40.6)**	**60 (70.6)**	**0.29 (0.12–0.66)**	**0.003**	0.48 (0.08–2.94)
Proximal diverticula	**8 (25)**	**41 (48.2)**	**0.36 (0.14–0.89)**	**0.026**	0.44 (0.06–3.37)
Blood in terminal ileum on colonoscopy	**8 (44.4)**	**7 (15.2)**	**4.46 (1.3–15.24)**	**0.017**	**5.04 (1.25–20.32)**
Blood in the colon lumen	13 (40.6)	22 (25.8)	1.95 (0.83–4.61)	0.124	
**Medication use on admission**					
Aspirin	19 (59.4)	39 (45.9)	1.72 (0.76–3.93)	0.195	
Coumadin	13 (40.6)	23 (27.1)	1.84 (0.78–4.33)	0.159	
Nonsteroidal anti-inflammatory drug	3 (9.4)	9 (10.6)	0.87 (0.22–3.45)	0.847	
**Laboratory values**					
Hemoglobin, g/dL, mean (SD)	8.86 (1.27)	8.34 (1.39)	0.73 (0.52–1.02)	0.065	0.72 (0.41–1.24)
Platelets, (10^9^/L), mean (SD)	197 (68)	204 (88)	0.99 (0.99–1)	0.689	
BUN, mg/dL, mean (SD)	26.3 (20.9)	20.2 (15.7)	1.02 (1–1.04)	0.113	
Creatinine, mg/dL, mean (SD)	1.67 (1.4)	1.65 (1.9)	1.01 (0.8–1.27)	0.965	
INR, mean (SD)	1.53 (0.73)	1.42 (0.47)	1.38 (0.61–3.12)	0.441	

(BUN: blood urea nitrogen, INR: international normalized ratio)

## Discussion

In this retrospective, two-center study including individuals presenting with overt GIB and suspected small bowel bleeding with normal upper and lower endoscopy, we identified a primary endoscopic factor associated with positive VCE, namely the presence of blood in the examination of the terminal ileum. Currently, the American College of Gastroenterology guidelines for LGIB recommends examination of the terminal ileum based on very low-quality evidence [2]. Our study provides additional evidence for the value of an examination of the terminal ileum in individuals presenting with overt GIB because the presence of blood visualized in the terminal ileum was a strong and significant predictor of positive VCE. Secondly, we identified diverticula as inversely related to a positive VCE and therapeutic intervention in univariate analysis. Differentiation of acute small bowel bleeding from an overt LGIB source from diverticular hemorrhage can be clinically challenging. While positive identification of diverticular bleeding has been associated with rapid bowel purge protocols [9], authentication of diverticula as a definite source of bleeding can fall to 10–20% as a source of LGIB [10]. In a population-based study from a large healthcare maintenance organization, while diverticular bleeding was cited as the most common source of LGIB, as many as 87% of the diagnoses for diverticular bleeding were presumptive rather than definitive [11]. When non-bleeding colonic diverticula are identified on colonoscopy, there is often a question as to whether to proceed with evaluation of a small bowel bleeding source by VCE. Here, we provide evidence that the presence of diverticula on colonoscopy is associated with a negative examination of the small bowel by VCE as well as therapeutic procedure to the small intestine, albeit only in univariate analysis.

VCE has become the recommended first-line evaluation for bleeding disorders of the small bowel. When compared to other imaging modalities, VCE has a reported higher diagnostic yield (25–50%) when compared to small bowel radiography (yield 3–20%), push enteroscopy (3–30%) or angiography (5–15%) [12]. Subsequent studies have demonstrated higher diagnostic yield in ongoing overt bleeding compared to individuals with a history of overt bleeding or with iron deficiency anemia [13]. Individual risk factors for positive VCE findings have included: inpatient status and male sex [12, 14], increasing patient age [15] and early inpatient VCE performance [16].

A single previous study has assessed for risk factors of small bowel bleeding in the setting of an overt presentation and negative endoscopic examinations [17]. Age greater than 65 years, anticoagulant use, antiplatelet use and NSAID use were predictive of positive findings on VCE. Endoscopic risk factors were not assessed. While our study did not identify antiplatelet or anticoagulant therapies on admission as associated with positive VCE, differences in inclusion criteria could account for the differences in findings. As endoscopic features were not presented, fewer individuals with colonic diverticula may have been included in the study by Katsinelos et al, which accounts for the lower diagnostic yield in our study as well as identification of diverticula as inversely related with positive VCE.

Identification of early predictors of overt small bowel bleeding allows for appropriate endoscopic examinations and resource utilization. Outside of predictive factors for a positive VCE, we separately report similar risk factors for therapeutic intervention among individuals undergoing VCE. Among individuals with a positive VCE, 80% underwent therapeutic intervention that is similar to previously reported small bowel intervention rates (66.6%) [18]. In the present study, diverticula on preceding colonoscopy were inversely associated with the performance of a therapeutic intervention. Furthermore, blood on terminal ileal examination was also positively associated with therapeutic intervention both in univariate and multivariate analyses.

The primary strength of this study includes the identification of endoscopic risk factors for a subsequent positive VCE in overt GIB. We expand previously identified clinical risk factors and focus on a single presentation, namely overt bleeding. All VCEs were performed as inpatient examinations and median time to VCE was 2.2 days. Therefore, our study allows for an accurate diagnostic yield as time to VCE was previously found to be predictive of positive findings [17]. Secondly, we provide evidence to support guideline recommendations to examine the terminal ileum on all colonoscopies performed for overt LGIB. Differentiation of acute small bowel bleeding from LGIB based on endoscopic examination of the small bowel has the potential to decrease resource utilization and reduce hospitalization duration in presentations with overt LGIB.

Our study is not without limitations. The sample size is moderate, therefore limiting the number of positive VCE examinations and our ability to create predictive models. As only 54.7% of the cases had examination of the terminal ileum, multivariate analysis is restricted to the cases that underwent examination, limiting our power to identify more independent predictors. Secondly, the diagnostic yield of VCE in this study was 29.9% falling between the previously reported 92.3% for active overt bleeding and 12.9% for those with a prior episode of overt bleeding [13]. Invariably, individuals with LGIB secondary to diverticular bleeding were included as suspected small bowel bleeding cases as diverticula were inversely related with positive VCE. Therefore, this study adds to the literature supporting the use of inpatient VCE in active overt bleeding, while limiting VCE utilization in those with prior overt bleeding and potential alternative sources of overt bleeding. Finally, the decision to proceed with VCE after negative upper and lower endoscopy was determined by the treating physician and was not performed routinely for all patients as part of this study. This could have biased our results in favor of patients who were more likely to have a small bowel bleeding source.

In conclusion, our study highlights the importance of identifying individual risk factors for positive VCE for overt GIB. We find supportive evidence for intubation and examination of the terminal ileum in overt LGIB as visualized blood is associated with a positive VCE and subsequent therapeutic intervention. We further associate alternative sources of LGIB with a decreased rate of positive VCE, thereby questioning the utility of VCE in clinically suspected diverticular bleeding. Future studies should continue to establish clinical predictive factors for positive VCE in identified clinical scenarios in order to guide appropriate diagnostic evaluations and resource utilization.

## Abbreviations

VCE: video capsule endoscopy
